# Qualitative Insights from the Osteoporosis Research: A Narrative Review of the Literature

**DOI:** 10.1155/2016/7915041

**Published:** 2016-11-22

**Authors:** A. E. Bombak, H. M. Hanson

**Affiliations:** ^1^Community Health Division, Central Michigan University, 2219 Health Professions Building, Mt. Pleasant, MI 48859, USA; ^2^Department of Community Health Sciences, Cumming School of Medicine, University of Calgary, TRW Building, Room 3D10, 3280 Hospital Drive NW, Calgary, Alberta, Canada T2N 4Z6; ^3^Seniors Health Strategic Clinical Network, Alberta Health Services, FMC, South Tower, Room 1103, 1403 29th Street NW, Calgary, Alberta, Canada T2N 2T9

## Abstract

*Purpose.* Much of the research on osteoporosis has been generated quantitatively. However, the qualitative osteoporosis literature provides valuable information on patient and clinician experiences and perspectives, informing the design and implementation of health research and healthcare services. To identify knowledge gaps and inform the design of future qualitative research, a narrative review was conducted to consolidate and synthesize the existing insights available within the qualitative osteoporosis research.* Methods.* Search terms reflecting the domains of osteoporosis and qualitative research were entered into the Scopus database to generate a comprehensive survey of qualitative research in the area of osteoporosis. Articles were thematically analysed and the results are presented in the form of a narrative review.* Results.* Forty-four articles were included in the narrative review. Qualitative research in the field of osteoporosis research can be summarized by 3 thematic areas: the meaning of osteoporosis for patients and the public, the lived experience of an osteoporosis diagnosis, and the programmatic approach to osteoporosis prevention and treatment.* Conclusions.* Qualitative studies provide clinically valuable insights in how osteoporosis is conceptualized and managed and programmatic aspects of osteoporosis treatment. The findings of this narrative review suggest the need for balance between presenting osteoporosis as a serious health condition and producing unwarranted anxiety and inactivity so as to ensure the best possible outcomes for individuals with osteoporosis.

## 1. Introduction

Osteoporosis is “a progressive systemic skeletal disease characterized by low bone mass density and microarchitectural deterioration of bone tissue, with a consequent increase in bone fragility and susceptibility to fracture” [1]. Globally, a third of women and 20% of men over 50 will experience osteoporosis [2]. Fractures can cause acute and chronic pain, deformity, diminished quality of life, disability, loss of independence, nursing home admission, and death [3–10].

The field of osteoporosis research is substantial. Major lines of inquiry span decades and include multiple disciplines. Research includes randomized clinical trials on, for example, secondary care prevention strategies [11–13] and patient decision aids quantifying care gaps [14, 15] and identifying at-risk groups [16]. However, while research on osteoporosis has proliferated, the field is dominated by quantitative research. Insights into individuals' experiences, motivations, behaviours, and perceptions are less common in this literature and can only be provided by the in-depth tools of qualitative research [17]. Qualitative research can provide insight into social meanings and behaviours from the perspectives of affected groups, explore little-understood phenomena, and help develop new theories [18]. For example, qualitative research can allow the field to move beyond numeric descriptions of individuals with fractures taking up osteoporosis risk screening opportunities to explore individuals' perceptions of future fracture risk, the reinforcing or constraining factors associated with osteoporosis investigations, and the decision-making constructs important to the design and implementation of patient-oriented risk screening and healthcare programming. A first step to rectifying gaps in the osteoporosis literature is a thorough assessment of the evidence currently available. Thus, this article seeks to fill this first step by presenting a comprehensive narrative review on osteoporosis-related research utilizing qualitative methods.

## 2. Materials and Methods

In advance of conducting a qualitative substudy within a larger program of research on secondary fracture prevention, a narrative review of the literature was conducted to identify and synthesize osteoporosis-related findings generated by qualitative approaches [19]. Literature was sought that crossed the conceptual domains of osteoporosis and qualitative methods. Multiple search terms under each domain were included (Table 1). Various iterations were entered into the Scopus database in July 2015. Hand searching of reference lists of relevant articles was also conducted. The broadest search terms osteoporosis and qualitative produced over 600 articles. More specific search criteria were used, that is, specific qualitative method and osteoporosis, to identify further articles. Restrictions were not placed based on date of article publication although irrelevant journals were discarded as the search progressed. Only English articles were reviewed. Articles that applied qualitative methods to understand patient, public, and provider perspectives on osteoporosis and its care were included in the narrative review and thematically analysed. The intent of this review is to elucidate common themes covered in qualitative osteoporosis research, not necessarily to present a comprehensive and systematic review of all published qualitative osteoporosis literature.

## 3. Results and Discussion

The studies included in the narrative review are summarized in Table 2. In total, 44 articles were included, representing significant global breadth by country of origin (see Table 2). Qualitative research in the field of osteoporosis research could be described with three thematic areas: the meaning of osteoporosis for patients and the public, the lived experience of an osteoporosis diagnosis, and the programmatic approach to osteoporosis prevention and treatment. Each area of qualitative research focus is described, in turn, below.

### 3.1. What Does Osteoporosis Mean for Patients and the Public?

#### 3.1.1. Metaphors and Cultural Models

Some authors have used qualitative techniques to explore how patients and the public understand osteoporosis and the risk of osteoporosis. Results often suggest a high level of confusion regarding the severity, personal relevance, and prevention and treatment of osteoporosis. Reventlow and others studied the metaphors used by Danish women in describing osteoporosis and their conceptualizations of osteoporosis and risk and are the few scholars who considered embodiment and osteoporosis [20, 21]. Descriptions suggested a lack of trust in one's body and a view of the osteoporotic body as nonnormative and relied on metaphors of destruction and frailty [21]. A focus on physical characteristics or appearance for assessing osteoporosis risk has been identified in other samples of older Danish women [22].

Osteoporosis was alternatively depicted as a natural by-product of aging, or as a serious disease, possibly amenable to prevention [20]. Oscillating between the seriousness of the disease and inconsequentiality, participants reported osteoporosis as catastrophic or irrelevant. Participants in some studies viewed osteoporosis as not personally pertinent [23], while others viewed it as inducing anxiety and preventive action, or in terms of its most dire, life-altering consequences [20, 21, 23]. Confusion was also seen concerning the effects of hormone therapy. Osteoporosis risk messaging is problematized by the authors of these studies who suggest it may produce anxiety among the well and a denigration of aging.

Similar confusion around osteoporosis is seen in other global contexts. Among Iranian-Australians, confusion was expressed regarding causes, risk factors, and treatment of osteoporosis. Culturally specific meanings of osteoporosis were expressed. Worries of retaining independence and the limited time available to consult with physicians were commonly cited [24]; however, patients also worried about not being able to seek out medical attention in their first language. Iranian women were concerned that emotional repression (silent culture) may contribute to osteoporosis [24]. Women expressed concern over sun exposure and appropriate fitness venues [24, 25]. In Turkey, osteoporosis risk was connected to menopause, wearing traditional clothing and praying. This is suggestive of the sociocultural valence allayed upon presumably clinical, objective conditions [25].

#### 3.1.2. Clinical Confusion

Understandings of bone health among Canadian patients with fractures in more clinical contexts have been extensively explored [26–29]. Patients do not connect underlying bone health to the resultant fractures, which are perceived as freak, traumatic accidents [26, 29]. A conceptualization of fractures as freak events has been reported by other authors (e.g., [22, 30, 31]). This dissociation had important impacts on medication adherence. While close to 2/3 of patients were taking medication, adherence was related to whether patients perceived their fracture as unrelated to underlying bone health. In turn, belief in the relationship between fractures and bone health was predicated on whether the fall occurred during more mundane circumstances [29].

Bone mineral density (BMD) testing is the primary test for the clinical assessment of bone mass and the subsequent diagnosis of osteoporosis [32]. Miscommunication and misinterpretation issues were evident in patients' understandings of BMD tests, although this did not necessarily impact adherence [27]. Diagnoses of osteopenia, the low bone mineral density precursor to osteoporosis [33], were particularly liable to misunderstanding. Such results tended to be minimized and viewed as not serious or inaccurate. Patients also, often erroneously, considered a lack of results to be indicative of good news. In general, patients did not describe their bone health in standardized ways, suggesting that more uniform communication of BMD results must be undertaken with fracture patients [27]. Inconsistencies were also evident in (mostly female) Canadian patients' perceived messages about bone health from various providers [28]. Compared to other providers from whom messages were rarely received, specialists were seen as more engaged in areas of bone health, up-to-date, and willing to consult with patients [28].

Cultural differences and metaphoric descriptors were frequent in the literature. Osteoporosis was alternatively viewed as a natural by-product of aging or a life-altering, catastrophic diagnosis, which impacts frail older women. Clinically, patients are confused by the connection between bone health and fractures and receive nonstandardized messages concerning osteoporosis that exacerbate confusion.

### 3.2. How Do Patients Live with Osteoporosis?

Coping strategies for osteoporosis have been explored qualitatively by some authors. Patients adopt multiple approaches for coping with osteoporosis. Strategies included discounting the everyday relevance of osteoporosis, by emphasizing their own capacities or denying its impact; focusing on living day-to-day, either by adopting a hopeful, positive attitude or a sense of resignation; limiting physical activity in fear of falling; and relying on social contacts, professionals, painkillers, or nonpharmacological aids and devices [31, 34–37]. Often, these approaches are affected by individuals' self-concept, resources, and stressors [35, 37]. Some patients become strong self-advocates for their care and engaged in related health behaviors, research, and interactions. However, others found it difficult to adhere to treatment regimes, despite acknowledging the importance of bone health [31].

Maintaining independence has been revealed to be of especial importance to patients with osteoporosis, and fractures can have profound effects on wellbeing [30]. Among Swedish geriatric rehabilitation patients, three areas of activity were particularly relevant for life satisfaction: going out of doors, social interaction, and care “close to the body.” Participants valued being able to persist in former activities in familiar settings [38]. Swedish, female patients with osteoporosis described maintaining independence as central to health-related quality-of-life [34]. Constant back pain was a threat to this independence, as was anxiety, self-image issues, and bodily limitations in carrying out everyday, social, and meaningful activities [34]. Maintaining independence was also of paramount concern for American, rural, older female patients with osteoporosis, who also spoke of challenges in shifting caregiver roles in families and anxiety over coping with the manual demands of farm life while suffering pain, functional limitations, and the possibility of falls [39].

Nielsen and colleagues conducted one of few studies on men's experiences of osteoporosis. Focus groups were undertaken with Danish men with osteoporosis [40]. The importance of maintaining a hegemonic masculine identity was highlighted by participants by being physically active and strong, only seeking healthcare as a last resort, and engaging in usual activities, traditional gender roles, and occupations. Osteoporosis often challenged these roles. Participants also felt feminized by the condition and its frequent attribution as a “female disease.” Participants with comorbid or more severe conditions, or those whose spouses suffered from such conditions, reconceptualised masculinity to incorporate more proactive health consumer behaviours [40].

Patients cope with osteoporosis in a multitude of ways, often reflecting their own self-concept; available social and capital resources; and views on health and aging. Particular value was placed on maintaining independence, dignity, and existing relationships and activities. Men must also negotiate, when coping with osteoporosis, its connotations as a female disease.

#### 3.2.1. Medication Adherence

Patients with osteoporosis express considerable confusion and ambiguity regarding medication, dosages, and supplements [26, 41]. Troublingly, this can persist despite participating in a standardized osteoporosis screening program, where education, written instructions, and direct care referrals were received [26]. Many researchers have explored reasoning and attitudes underlying medication adherence. Central to these issues appears to be relationships with primary providers [41–43]. Having a trusting relationship with a clinician could ease making a decision to take medication; however, current adherence did not necessarily preclude future cessation of medication use among Canadian patients with osteoporosis [42]. If concerns about medications use were not alleviated by practitioners, individuals often resorted to other sources of information (other providers, friends, family, and pamphlets), as did participants in other studies (e.g., [44]). Worryingly, these external consultations could result in more negative appraisals of these medications [44]. Similarly, in focus groups, American patients with osteoporosis cited use of external research and emphasized primary provider, self-image, and medication confusion issues as affecting their likelihood of adherence [45].

Perspectives of adherence held by health providers were also described in the literature. Providers tended to focus on system issues, such as interprovider characteristics; the asymptomatic nature of osteoporosis; and patient characteristics, such as use of external information sources. Common ground with patients was found in areas of side effects, costs, importance of trusting provider-patient relationships, and need for memory aids [45]. These more objective criteria for adherence were not replicated among English and Danish patients with osteoporosis. These patients were largely compliant with medication regimes and characterized their reasoning in terms of guilt and fear, rather than issues of side effects or cost [35]. Fear was also a motivating factor among Canadian postmenopausal patients with osteoporosis [44], although presumed and experienced benefits of medication adherence were also reported as a motivator [44]. English female patients with osteoporosis were cited as prioritizing fall prevention over medication adherence but recommended the use of pictures of bones as an adherence technique. The patients often illustrated osteoporosis via emotionally resonant drawings of spine curvature and height loss [41]. The studies above point to another major knowledge gap in the literature, the role of emotion in living with osteoporosis and its relevance in clinical decision-making. Furthermore, the use of other methods, such as participant observation, would be highly valuable in exploring adherence.

Perspectives on adherence may be affected by race. Neuman and colleagues report that Black Americans were less likely to express a willingness to have fracture surgery compared to White Americans [46]. They were also characterized by greater levels of healthcare distrust, lower education levels, greater comorbidities, and fewer prior surgeries compared to their White counterparts. In qualitative freelist exercises, Black participants identified different concerns than White participants. Importantly, distrust of physicians, particularly in primary care physicians' knowledge and engagement in bone health, and medications has been found in numerous populations [41, 44, 47, 48].

Similar themes were identified from focus groups on medication adherence among a racially diverse sample in the United States [48]. Calcium was considered a cheap and safe alternative to potentially riskier and expensive medications, and participants considered themselves to be not at risk for osteoporosis, in part, due to race, and this affected medication adherence. Both of these findings parallel views among female Canadian patients with osteoporosis [44]. Participants in both studies also stated a preference for once-a-week medications, although this was cost prohibitive for the American sample [48]. Osteoporosis was considered less severe than heart disease and cancer. Distrust of physicians' competence, the unnaturalness of medications, and risk of side effects also affected adherence [48]. For those who did consider fractures to be a major concern, adherence was likely. Adherence was also likely amongst those who trusted medications and experienced benefits in functioning or symptoms from taking the medication. However, not experiencing symptoms could also motivate medication cessation [48].

Brod and colleagues also explored adherence issues and osteoporosis treatment [49]. They conducted data collection with physicians who had prescribed self-injectable osteoporosis treatment and the patients to whom these injections had been prescribed. Physicians' familiarity with the medication was a major issue in prescribing, and whether they felt data was sufficient in areas such as side effects, efficacy, and long term effects. Concerns were also raised regarding costs, reimbursement issues, and availability of staff for training and followup. Patients who had experienced previous fractures and were worried about future issues chose the medication. Severe side effects and perceived efficacy affected persistence of the medication, and this could be mediated by high initial expectations [49].

Nonpharmacologic fracture risk prevention strategies were also described in the literature. Canadian older adults at high risk for future fractures described a variety of methods for protecting themselves from future fracture [36]. Mostly, these strategies centred on being careful and modifying existing environments to prevent falls. Other efforts involved diet, exercise, supplements, and mobility aids and devices [36]. Interestingly, the use of nonpharmacological strategies was independent of medication adherence. Participants were aware of their fracture risk status, suggesting to the authors that knowledge in this area may be filtering out to the general public.

Overall, patients and their providers described complex and contingent reasoning related to medication adherence. Prolific researchers in the field, Sale and colleagues, ponder the utility of adherent/nonadherent labels given the fluid and adaptive negotiations undertaken by patients with respect to osteoporosis medication use [42]. Also of note is that while the asymptomatic nature of osteoporosis is highlighted as a cause for nonadherence, patients in these studies frequently recount pain [26, 34, 41, 50], which is often dismissed as nonosteoporotic by researchers, but may be an important factor to consider with respect to adherence. Indeed, pain is little explored in-depth in this literature, despite being of obvious salience to participants. While issues of cost and convenience were relevant to some patients, in general, many patients were distrustful of medications and required reassurance as to their safety, side effects, and efficacy. If relationships with providers failed to convince patients as to the advantages of adherence, they turned to alternate sources or rejected medication. Some patients were motivated more by fear of consequences of nonadherence, while others disclosed belief in the benefits of medication on functioning and symptoms.

#### 3.2.2. Patient Engagement

Some authors focused on patients' engagement in care and attitudes toward involvement in clinical encounters. In focus groups and interviews, older Danish women described their views on osteoporosis screening [22]. Help seeking behavior was perceived as a moral responsibility, but screening could be rejected by those who felt they were at low risk for osteoporosis. Osteoporosis screening was not considered to be as important as cancer screening; however, most participants did undertake screening, while acknowledging concerns over labelling, medicalization, and medication effects [22].

The health-consumer behaviours of members of a national Canadian osteoporosis group have been explored by Sale and colleagues [47]. While individuals were recruited under the assumption that they would be effective health consumers, the authors detected that most participants could be categorized as at the extreme ends, either exhibiting many or few health consumer behaviours. Participants were categorized as either having few effective behaviours or more effective consumers behaviours, based on their communication with clinicians and care involvement. Participants with more effective behaviors sometimes expressed a lack of trust in primary care physicians and viewed them as largely passive in their care. These more effective participants considered themselves bad patients due to their self-advocacy. They were also less frequently compliant to medication regimes and often researched, switched, and ceased medications. Patients with more fractures reported more effective behaviours. The authors note that while the focus was on categorizing patients as “more” or “less” effective consumers, placing the onus of care on patients may exacerbate stigma [47].

A study in the UK also demonstrated the complex, real-life manifestation of “patient engagement” that reflects idiosyncratic preferences for control in clinical encounters and the moralistic dimension that patient engagement may assume. Focus groups were conducted with patients to evaluate Decision Aids (DAs) designed for osteoporosis and a number of other conditions [51]. Patients appreciated the information contained in the DAs as a possible launching point for greater involvement. However, not all patients wished to assume the “burden” of decision-making and preferred the more paternalistic approach to patient care. The authors conclude that DAs may be useful, as long as it is made clear to patients that physicians are not necessarily relinquishing responsibility [51].

Providers' perspectives on patient engagement in osteoporosis care have also been explored. Specialists involved in treating schizophrenia and osteoporosis in the UK and the USA were interviewed on patients' influence on treatment [52]. Notable differences were found between osteoporosis and schizophrenia patients' approaches. Osteoporosis patients were more actively involved than patients with schizophrenia and relied on different sources of information, such as the internet, rather than solely on family or friends. Physicians could be resistant to patients' own research activities or view these as time- consuming or biased by media. Overall, physicians described patients as influencing prescribing in a variety of ways: from their description of symptoms, side effects, and conditions, their stated preferences and refusals, and ultimately their medication adherence [52].

While patient engagement is increasingly advocated, its manifestation and implications in osteoporosis care suggest a more complicated reality. Patients view involvement in care, clinical decision-making, and screening as a moral responsibility, and they experience variable willingness to adopt these responsibilities. Patients' views on engagement and their behaviors varied across conditions. How a patient handles their osteoporosis care may differ from approaches to care for other conditions. Greater involvement, including more questioning behaviours and independent research, may produce lower adherence to prescribed medication regimes. Some providers demonstrate ambivalence about patient engagement, urging greater patient education but also finding engaged patients to be time-consuming and potentially biased [52]. More research is needed to elucidate these issues and preferences, particularly as patient-centred and patient-engaged and activated care are increasingly promoted and may not be preferred by patients in all situations.

### 3.3. What Are Patients' and Providers' Views of Programs in Place for Osteoporosis?

#### 3.3.1. Patients' and Caregivers' Views

Patient perspectives on specific care programs have been explored through qualitative research. Some scholars have focused on the multiple transitions that patients undergo following hip fractures [53, 54]. Identified themes included the importance of families as advocates for older relatives and issues of comorbidity and complications. Conflict and distrust emerged when participants felt that they were experiencing avoidable complications and the associated suffering, when they felt they were not integrated into their own care or when uncertain about processes and the roles of their providers. Transitions could involve all of medication errors, policies inhibiting individual care, communication issues, and fragmentary care [53, 54].

Potential resources for patients with osteoporosis have been reported following qualitative inquiry. Navigation within the recovery process of Canadian patients following hip fracture has been explored [55]. Similar to other studies, these participants described the need for more information on hip fractures and recovery, and the difficulty of questioning healthcare providers. One recommendation was the dissemination of a recovery map to help patients understand the recovery process. Other recommendations for patients included accepting offered help, maintaining a positive attitude, and moving more. Online resources for caregivers of patients following hip fracture have been developed and evaluated [56]. Online discussion boards were moderated by a nurse who used self-efficacy-informed theory to provide encouragement to caregivers. Discussion posts revolved around types of care provided by caregivers, fracture prevention strategies employed by caregivers, and stress-coping mechanisms. Themes within these discussions centred on the psychosocial burden of being a caregiver and potential lack of knowledge in this area, patients' expectations and transition issues, and the role of clinicians in therapy and utility of the internet program. Interestingly, discussions within this forum suggested that caregivers' concerns could potentially lead to discouraging beneficial physical activity. The discussion boards served the added purpose of educating caregivers about their own osteoporosis-related health needs [56].

Another internet-based fracture prevention program was evaluated using mixed methods [57]. The quantitative analysis suggested little improvements in all but knowledge and beliefs, that is, not behaviours. However, qualitative appraisals were more positive, speaking to the valuable nature of the program in strategizing goal setting, and nutrition and exercise training. Offering flexible tutorial lengths, use of other media, and multiple tutorials were all recommended by participants [57]. In addition to these externally produced online resources, more research is needed on patient-generated fora for individuals with osteoporosis and the potential establishment of virtual communities.

Further emphasizing the value of exercise instruction for osteoporosis patients, an in-person intervention featuring supervised group back muscle training was assessed by Qvist and colleagues [50]. The female participants' responses were generally positive, with a particular focus on reduced pain, improved sleep and increased mobility, strength, confidence in appearance, and physical activity. Participants described heightened awareness of their bodies through the exercises and the utility of enhancing strength and mobility. The importance of the program's physiotherapist's expertise in appropriate exercises for this population was highlighted. Social support benefits were also produced via the group environment. This contrasts with more mixed findings from Danish and English osteoporosis patients [35]. These patients reported that group-based education programs could be helpful and supportive environments but could also be disheartening in exposure to others' sicker than oneself or those seemingly competing to be the sickest in the group [35].

Overall, participants' and their caregivers' experiences of osteoporosis prevention and treatment are characterized by complex, sometimes problematic transitions and care-disruptions that can be complicated by patient confusion, lack of knowledge of recovery and processes, and feelings of exclusive from clinical relationships. The importance of provider expertise and appropriateness of content was highlighted in prevention-related research. Evaluations suggest that prevention programs produce benefits in knowledge and awareness, and those that incorporate in-person exercise may also benefit physical outcomes and behaviours.

#### 3.3.2. The Provider View

Providers' perspectives on osteoporosis programming have also been explored. Hand-offs and transitions have been explored in depth in Canada. An ethnography of physiotherapy hand-offs in Ontario revealed the importance of inter-professional handoffs between physicians and physiotherapists. Similar to other studies (e.g., [53]), the importance of family caregivers also emerged [58]. Clinician-patient hand-offs were characterized by unidirectional transmission of knowledge, little post-discharge provider responsibility for patients, and difficulties scheduling patient education, which often resulted in frustration and anxiety. Physiotherapists often relied on contacting patients' surgeons rather than previous physiotherapists to clarify patient information. Caregivers and clinicians often had to navigate multiple sources of data, including paper notes concerning their patients [58]. Communication issues were also highlighted in a strength-based exploration of hip fracture transitions in British Columbia [59]. Success in transitions was defined as focusing on processes and outcomes. The importance of information, communication, adaptability, and a formalized feedback loop was emphasized. Concerning outcomes, maintaining patient autonomy was emphasized, and care pathways were frequently referenced, but sometimes in critical ways [59].

Beyond provider views of transitions across health system settings, health professionals' perspectives have also been sought regarding osteoporosis programming. As part of prototype development of a clinical decision support system for an osteoporosis screening tool, focus groups with general practitioners were conducted in Canada [60]. Practitioners expressed concerns about patients' understanding; technological and time, resources, and process issues [60]. Furthermore, there were concerns that osteoporosis screening might disrupt the actual intended reason for the visit. Patients evaluated a prototype and made suggestions to the content and format of the tool, but while most indicated comprehension of the results, patients stated they would discuss their results with their physicians [61]. In the area of secondary prevention, Drew and colleagues explored the implementation of secondary hip fracture prevention programs in 11 English hospitals using Normalization Process Theory and semistructured interviews with health care providers including fracture prevention nurses, orthogeriatricians, and service managers [62]. In general, the services were considered feasible and relatively simple to implement. Participants were largely enthusiastic about the programs and demonstrated this through dedication to transparency and the paperwork and communicative processes affiliated with the program, although the efficacy of such communication was sometimes doubted [62]. The importance of multidisciplinary meetings and space issues, including cooperative spaces and physical proximity to the program, was highlighted. The value of a dedicated fracture prevention coordinator to relieve time constraints and unify multidisciplinary teams was also emphasized. Issues arose with respect to communication, attention to patients, equipment, and access for patients [62].

Generally positive attitudes respecting prevention were expressed regarding screening among older women and general practitioners in the UK [63]. Overall, screening did not substantially affect anxiety or activity engagement. Results of screening helped produce preventive behaviours to reduce risk of fracture. Main concerns revolved around targeting and cost-effectiveness. Location of scans, fears of the magnetic resonant imaging machine, and costs of the scans were all raised as issues for implementation [63]. Regarding nutrition, Dutch healthcare professionals' perceived barriers to implementing a nutritional intervention for older adult hip fracture patients have been explored [64]. Issues identified by this work included a dearth of knowledge concerning and time for delivery of appropriate nutritional care. Noncontinuity of nutritional care as patients transitioned to alternate institutions, lack of patient-specific approaches, and the deficiency of nutrition care policies and stated goals were also identified as potential hindrances to the feasibility of a nutritional intervention [64].

Finally, provider knowledge and attitudes have been topics of qualitative exploration. Claesson and colleagues explored Swedish nurses' perceptions on osteoporosis and osteoporosis management [65]. Their findings suggest alignment with the views of osteoporosis patients (as reviewed above) in a number of areas. For example, Swedish nurses tended to prioritize fall prevention and rated their competence highly in this area. Also similar to patients was a discounting of the seriousness of osteoporosis; a lack of knowledge and confidence concerning osteoporosis treatment; and a distrust of osteoporosis medications [65]. Nurses also identified the inadequacy of existing procedures and resources but expressed a willingness to identify patients perceived to be at high risk, learn more, and work with other professionals [65]. Findings from Canadian [66] and Australian primary care physicians [67] may validate some patients' concerns (reviewed above) that primary care physicians are not knowledgeable or concerned with bone health. Australian practitioners considered osteoporosis potentially debilitating among older adults but of less importance than many other chronic conditions [67]. Focus groups revealed that Canadian primary care physicians found osteoporosis clinical guidelines excessively detailed, were confused regarding osteoporosis medication, and would like greater osteoporosis clinical knowledge [66]. Furthermore, these practitioners were unsure of older adults' willingness to take more medication [66]. Similarly, Australian general practitioners considered medications potentially cost prohibitive for older adults, although they did believe in their efficacy [67]. Canadian primary care physicians rarely sent patients for bone tests, particularly men and premenopausal women, and were dissatisfied with presentation of BMD results [66], but Australian practitioners' approaches to screening were more varied [67]. However, doubt over treating men was shared by both nations' practitioners [66, 67]. Somewhat contradictorily, Canadian physicians advocated for greater patient education but felt that the media prompted unnecessary patient demands that burdened already overfilled appointments dealing with complex issues [66]. The Australian practitioners' views on patients informed their treatment approaches. Some practitioners preferred lifestyle modifications and only resorted to medications when such behaviours were unachievable. Others immediately prescribed medications, based on the belief that patients were incapable of lifestyle changes [67].

To summarize, prevention and treatment programming in the area of osteoporosis are generally well-supported by patients and providers. Concerns revolve around resources, time limitations, communication, and navigating role responsibilities in team contexts. Research into nurses' and primary physicians' attitudes toward osteoporosis suggests that osteoporosis is not necessarily a prioritized condition in already overburdened care encounters, and practitioners require greater knowledge on screening guidelines, medications, and treating men.

Areas that appear underrepresented in the qualitative osteoporosis literature include the perspectives of men with osteoporosis and the investigation of differences in osteoporosis identification and management among individuals with cognitive decline. Further information would be beneficial to explore how men experience osteoporosis which could provide healthcare professionals with insight into how they could better address the needs of male patients with osteoporosis. Individuals with cognitive impairment are also rarely included in studies, exacerbating the existing care gap of this vulnerable population. These observations may present two topics ripe for further investigation using qualitative approaches. Also of significance is the role of affect and pain in making clinical decisions concerning osteoporosis. Furthermore, there is little engagement with embodiment theory, which might be highly relevant to exploration in these areas, and the use of participant observation to contextualize the lives of patients in adherence studies.

### 3.4. Limitations

The review is characterized by a number of limitations. Scopus is the largest database of peer-reviewed literature including fields of technology, science, medicine, social sciences, and arts and humanities. However, use of an additional database for searching may have produced more comprehensive results. Conference abstracts and articles not in English were not included in the review. The search terms utilized may not have detected all relevant studies; however, this review presents an overview of the major foci of qualitative studies conducted in the area of osteoporosis. Given the breadth of articles reviewed, it is likely that additional articles would have concentrated on similar content areas.

## 4. Conclusion

This narrative review has clarified the extent and focus and consolidated the major findings of qualitative osteoporosis research. The major foci of this research have been on understanding the meaning of osteoporosis for patients and the public, the lived experience of an osteoporosis diagnosis, and the programmatic approach to osteoporosis prevention and treatment. This work adds to the clinically oriented literature on some of the underlying reasoning for particular outcomes in program implementation and patients' knowledge and adherence. Qualitative researchers have provided numerous insights into cultural understandings of osteoporosis, medication adherence, coping strategies, and programming.

Collectively, the studies included in this narrative review suggest a need to move the public's perceptions away from an appearance-focused assessment of osteoporosis risk and strike a balance between presenting osteoporosis as a serious health condition and producing unwarranted anxiety and inactivity among healthy older adults. More qualitative research is needed, particularly among men with osteoporosis and those with cognitive decline and also in the areas of patients' experiences of pain and affective considerations of medication use. Clinically, a greater understanding on what constitutes ideal patient-provider partnerships is necessary, as is an elucidation of medication adherence as an in-flux negotiation between patients and clinicians, in order to ensure the best possible outcomes for individuals with osteoporosis.

## Figures and Tables

**Table 1:** The search domains and associated terms used to conduct the narrative review.

Osteoporosis domain	Qualitative domain
Osteoporosis	Qualitative
Hip	Interview
Fracture	Focus group
Care	Ethnograph^*∗*^
Patient	
Adherence	
Engagement	

*∗* denotes the use of a wildcard search operator to retrieve variations of the word stem.

**Table 2:** Data extraction of evidence included in the narrative review.

Author (Year)	Geography	Purpose of the study	Methods	Sample	Key findings
Åberg, 2008 [38]	Sweden	Explore preferences of elderly care regarding activity-related life space and life satisfaction	Interviews; general motor function assessment; thematic framework analysis	Geriatric rehabilitation patients (80–94 YA^a^; *n* = 15)	Importance of socializing, going out of doors, continuity of activities in familiar settings, and body-related activities identified.

Baheiraei et al., 2006 [24]	Australia	Explore understandings of risk factors and barriers to osteoporosis prevention and control	Interviews; focus groups; group discussions; thematic analysis	Iranian-Australians (35–70 YA; *n* = 37)	Many culturally-specific misunderstanding and obstacles concerning osteoporosis prevention.

Besser et al., 2012 [41]	England	Explore patients' perceptions of osteoporosis and treatment	Interviews; drawings; self-regulation model	Osteoporosis/osteopenia patients (avg. age 69; *n* = 14)	Patients understand of osteoporosis but not medication and risk. Pictures elicit emotional responses.

Bhavnani and Fisher, 2010 [51]	UK	Explore patients' views of decision-aids	Focus groups; thematic content analysis	Patients of numerous conditions (42–83 YA; *n* = 77)	Decision-aids valuable as conversation starters should not replace clinical decision-making.

Breedveld-Peters et al., 2012 [64]	Netherlands	Explore barriers and facilitators of implementing hip fracture nutrition intervention	Interviews; focus groups; triangulation	Healthcare professionals (*n* = 35)	Barriers include lack of knowledge, role clarity, communication, and standardization in care.

Brod et al., 2008 [49]	USA	Understanding patient and physician adherence issues of self-injectable osteoporosis medication	Interviews; focus groups; analyzed for themes and conceptual model development	Osteoporosis patients (42–88 YA; *n* = 22); physicians (*n* = 6)	Motivation, physician messages, side effects, and clinical profile affected patients' adherence and persistence. Physicians were affected by knowledge, patients' clinical profile, and resources for patient education.

Claesson et al., 2015 [65]	Sweden	Explore nurses' perceptions of osteoporosis management	Focus groups; thematic content analysis; triangulation	Nurses (*n* = 13)	Barriers: insufficient knowledge/time to treat osteoporosis, a low priority condition; distrust of bisphosphonates; opportunities: competence in fall prevention and collaboration; willingness to learn more and identify at-risk patients.

Drew et al., 2015 [62]	England	Understand how and why secondary fracture prevention services can be implemented	Interviews; Normalization Process Theory	Healthcare professionals (*n* = 43)	Highly workable and easily integrated due to planning, multidisciplinary meetings, and technology. Challenges in coordination with primary care, lack of resources, staff, and patient access.

Drieling et al., 2011 [57]	USA	Develop and explore internet-based fracture-risk intervention	Mixed method randomized clinical trial: questionnaires; focus groups; tutorial evaluation forms	Women (≥19 YA *n* = 121)	Improvements in knowledge: qualitative results suggest benefits in behavior not evident in quantitative results.

Emmett et al., 2012 [63]	UK	Explore acceptability of osteoporosis screening	Interviews; focus groups; thematic framework analysis	Women (70–85 YA; *n* = 31); general practitioners (*n* = 15)	Screening viewed positively and not a promoter of anxiety. Must be cost-effective.

Claesson et al., 2015 [65]	Turkey	Examining medicalization and conceptualizations of risk regarding postmenopausal menopause	Group and individual interviews, participant-observation; media analysis; situational analysis	Menopausal women (*n* = 43); clinicians (*n* = 21)	Menopause is a risky period leading to osteoporosis. Traditional lifestyles also produce osteoporosis risk.

Hallberg et al., 2010 [34]	Sweden	Explore health-related-quality-of-life and daily life effects of vertebral fractures	Interviews, inductive content analysis	Females who experienced vertebral fracture ~9 years ago (68–74 YA; *n* = 10)	Independence was highly valued and threatened. Pain, self-esteem, and social life were affected. Various coping mechanisms were deployed including social support, self-care, and personal meaning.

Hvas et al., 2005 [23]	Danish	Explore effects of knowledge of osteoporosis risks	Interviews; editing analysis style	Menopausal women (*n* = 17)	Confusion, anxiety, and uncertainty accompanied awareness of osteoporosis risk; some dismissed personal risk.

Iversen et al., 2011 [45]	USA	Describe and contrast providers' and patients' views on adherence	Focus groups; open coding	Osteoporosis patients (65–85 YA; *n* = 32); general practitioners (*n* = 11); nurse practitioner (*n* = 1)	Relationship with provide could affect adherence as could confusion, issues with taking medication, source of information, and satisfaction with clinician; self-image and psychological wellbeing affected by osteoporosis; physicians felt cost, structural barriers side effects, knowledge issues impacted adherence, recommended memory aids.

Jaakkola, 2007 [52]	USA and UK	Investigate physicians' view of patients' participation in treatment	Interviews; analyzed for themes	Specialist physicians (*n* = 20)	Patients do influence treatment based on their resources and preferences, efforts and actions, expectations, and role expectations.

Jaglal et al., 2003 [66]	Canada	Explore physicians' experiences and perspectives of osteoporosis and educational needs	Focus groups; constant comparative analysis	Family physicians (*n* = 32)	There was confusion over management. Tests were ordered but lacked rationale. Required greater clinical education.

Johnson et al., 2013 [58]	Canada	Examine information exchange during rural hip fracture transitions	Interviews, participant observation, record and policy analysis; focused coding and framework development	Hip fracture patients (>65 *n* = 11), healthcare providers (*n* = 24); caregivers (*n* = 8).	Information needed to be timely but had to navigate numerous sources to obtain information. Families often had difficulty obtaining information.

Kastner et al., 2010 [60]	Canada	Understand physicians' perceptions of an osteoporosis clinical decision support system	Progressive, iterative focus groups; open, axial, and selective coding	Physicians (*n* = 16)	Suggestions were made for modifying tool. Barriers included use of tablet device in waiting room, potential for patient confusion, concerns over extracting information from tool.

Kastner et al., 2010 [61]	Canada	Conduct a usability study of an osteoporosis clinical decision support system	Qualitative components: interviews; audio-taped one-on-one usability sessions; constant comparison analysis	Study 1: physicians (*n* = 11); study 2: patients (avg. 72 YA; *n* = 19); study 3 (avg. 73 YA; *n* = 8)	Patients found most components of the tool comprehensible and valuable to clinical encounters. Found questionnaire difficult to initiate. Physicians concerned over timing, workflow, and disruption of clinical encounter.

Lau et al., 2008 [44]	Canada	Explore factors influencing adherence and perceptions of adherence strategies	Focus groups; analyzed for themes; triangulation through member checking	Females taking osteoporosis medication (48–88 YA; *n* = 37)	Factors affecting adherence: beliefs regarding medications, importance of medication, and health; medication-specific factors; information exchange; and adherence strategies; memory aids, information, systems, and ongoing provider follow-up.

Meadows and Mrkonjic, 2003 [30]	Canada	Understand experiences and sequelae of midlife fractures in women and whether connections are made to underlying bone health	Interviews; crystallization/immersion	Female fracture patients 40–65 YA at time of fracture (*n* = 19)	Fractures produced pain and major life confusion. Connection between bone health and fracture was often confused and care was discontinuous. Women often devastated by osteoporosis diagnosis and felt they had been low risk.

Meadows et al., 2005 [31]	Canada	Explore women's perceptions and experiences of fracture	Interviews; immersion	Female fracture patients (40–65 YA; *n* = 22)	Three responses to risk: laissez faire approach, inconsistent adoption of change/knowledge, actively engaged in knowledge seeking/behaviour change.

Nahm et al., 2013 [56]	USA	Explore caregivers' caregiving experiences while using an online hip fracture resource centre	Content analysis of online discussion board postings	Caregivers of recovering hip surgery patients (*n* = 27)	Caregivers discussed types of care and coping strategies; fracture prevention strategies; themes included: recognition of clinicians, utility of program, caregivers' stress and lack of knowledge; care recipients' need for adjustment; desire for baseline status, and transition difficulties.

Neuman et al., 2013 [46]	USA	Explore racial variations in preferences for hip fracture care	Qualitative component: freelist exercises	Black and White geriatric medicine patients (*n* = 66; 69–79 YA)	Blacks and Whites differed in salient downsides of surgery. Whites more concerned with complications and surgical skills. Blacks more concerned with recovery time, inability to care for oneself, lack of success, and death. Pain and recovery time concern for all. Quantitative results: Blacks less favorable view of surgery.

Nielsen et al., 2011 [40]	Denmark	Examine how men handle osteoporosis in everyday lives	Focus groups; critical psychology analysis	Men with osteoporosis (*n* = 16; 51–82 YA)	Men concerned with maintaining masculine identity through maintaining strength, activity, and being proactive in treatment.

Nielsen et al., 2013 [35]	England and Denmark	Explore importance of osteoporosis knowledge on patients' everyday handling of osteoporosis	Interviews; participation observation; phenomenological meaning condensation; critical psychology analysis	Osteoporosis patients (*n* = 26)	Life conditions affect how osteoporosis is handled, everyday life influenced by handling of treatment, handling of osteoporosis information affected patient experiences and relationships.

Otmar et al., 2012 [67]	Australia	Investigate barriers and enablers affecting osteoporosis investigation and management	Focus groups; analytic/nominal comparison; thematic coding	General practitioners (*n* = 14) and practice nurses (*n* = 2)	Osteoporosis of less concern than other conditions. Unsure of guidelines regarding men and duration of treatment. Believed in bisphosphonate efficacy but worried about cost.

Popejoy et al., 2013 [53]	USA	Describe types of care transitions and problems experienced by hip fracture patients	Chart reviews and interviews	Hip fracture patients (68–97 YA; *n* = 21)	Patients experienced a median of 4 transitions. Families vital for advocacy and identifying problems. Care complicated by comorbid conditions. Patients desired faster recoveries and more aggressive treatment. Transition to skilled nursing facility experienced greater issues than transition to inpatient rehabilitation facilities. Common issues: delirium, depression, falls, urinary incontinence, pressure ulcers, and weight loss.

Qvist et al., 2011 [50]	Sweden	Investigate experiences of a back muscle exercise group for women with osteoporosis-related vertebral fractures and thoracic kyphosis	Interviews; content analysis	Participants in the back muscle group for women with osteoporosis-related vertebral fractures and thoracic kyphosis (*n* = 11)	Participants described physical, behavior, and psychosocial benefits from the group. Awareness and experiences of the body from the exercise (awareness of straightening the back and usefulness of increased strength and mobility) and social dimensions of the training (affinity and support and sense of trust and safety).

Reventlow and Bang, 2006 [20]	Denmark	Explore Danish women's ideas regarding osteoporosis and risk	Focus groups; meaning-centred analysis	Women (60-61 YA; *n* = 23)	Risk of osteoporosis assessed by appearance; vacillation between osteoporosis as product of ageing or preventable disease; women concerned with osteoporosis risk viewed it in catastrophic terms.

Reventlow et al., 2008 [21]	Denmark	Explore women's conceptions and models of osteoporosis risk	Focus groups; interviews; analyzed for metaphors; coded for schemata-based structures	Women born in 1936 who had heard of osteoporosis (*n* = 40)	Findings suggest a lack of trust in one's body and negative view of ageing. Osteoporosis is nonnormative and destructive. Commonest metaphor was a collapsing building. Imagery included porous bones, frail bodies, and collapsing backbones.

Roberto and Reynolds, 2001 [39]	USA	Explore functional and psychosocial consequences of living with osteoporosis	Focus groups; open coding for themes and patterns	Females with osteoporosis living in rural communities (53–89 YA; *n* = 21)	Main categories: describing history of identifying and diagnosing osteoporosis; changes in daily activities (functional abilities and social interactions and relationships); concerns and challenges (including self-concept, fears, and independence); coping interventions (pharmaceuticals, supplements, devices, and exercise); advice for other women with osteoporosis.

Rothmann et al., 2014 [22]	Denmark	Explore patients' patients' perspectives, experiences, and acceptance of the program	Interviews; focus groups; critical psychology approach; analyzed for themes	Women (65–80 YA; *n* = 52)	Limited knowledge of osteoporosis. Acceptance of screening affected by patients' overall life, experiences, and view of risk and preventive measures. Health-seeking perceived as moral obligation, whether or not screening accepted. Screening served valuable role in reassurance or elevating concerns.

Sale et al., 2010 [26]	Canada	Investigate understanding of osteoporosis and related care after osteoporosis screening and care	Focus groups; analyzed for themes	Fracture patients screened at an osteoporosis screening clinic (47–80 YA; *n* = 24)	Uncertainty common. Patients were ambiguous about the cause of their fracture (not linking falls to osteoporosis); osteoporosis's presentation as a disease (due to asymptomatic nature); BMD testing and results; and medication and supplements.

Sale et al., 2010 [27]	Canada	Assess patients' interpretations of BMD results and perceptions of bone health	Interviews; iterative, phenomenological analysis	Fracture patients with a previous BMD test (49–82 YA; *n* = 18)	A third of patients accurately recounted test results. Test results not related to medication adherence. Patients presumed (not necessarily accurately) that receiving no news was indicative of healthy status. Test results not taken seriously or viewed as accurate and these views were related to adherence.

Sale et al., 2011 [42]	Canada	Examine patients' experiences making osteoporosis medication decisions following a fracture	Interviews; phenomenological analysis guided by Giorgi's methodology	Fracture patients at high risk for future fracture (65–88 YA; *n* = 21)	Ease of decision affected by relationship with provider. Less sure participants sought outside information and were concerned over side effects. Decisions were subject to change.

Sale et al., 2014 [36]	Canada	Explore patients' nonpharmacological/diagnostic strategies for managing bone health/fracture risk	Interviews; phenomenological analysis guided by Giorgi's methodology	Fracture patients at high risk for future fracture (65–88 YA; *n* = 21)	Participants focused on being careful and altering perceived modifiable personal and environmental factors, exercising, altering diet, and using aids and supplements.

Sale et al., 2014 [47]	Canada	Examine members of an osteoporosis patients group members' behaviours and experiences managing bone health	Interviews; phenomenological analysis guided by Giorgi's methodology	Members of a national osteoporosis group who sustained a fracture ≥50 YA when not taking medication (51–89 YA; *n* = 28)	Patients were categorized as more or less effective health consumers. Over half were effective consumers based on interactions with providers for medications, tests, referrals and other behaviours.

Sale et al., 2015 [28]	Canada	Examine perceived bone health messages among members of an osteoporosis patient group	Interviews; phenomenological analysis guided by Giorgi's methodology	Members of a national osteoporosis group who sustained a fracture ≥50 YA when not taking medication (51–89 YA; *n* = 28)	Perceived messages were very inconsistent. Greater osteoporosis interest perceived in specialists. Other providers rarely relayed messages.

Schiller et al., 2015 [55]	Canada	Summarize patients' messages or advice for recovering from hip fractures and how these messages can be used in clinical communication.	Interviews; inductive coding	Patients ≥60 with a previous hip fracture and their caregivers	Three main messages helped recovery: seeking support, moving more, and preserving perspective.

Sims-Gould et al., 2012 [59]	Canada	Understand key elements of healthcare provider-perceived success in care transitions	Interviews; analytical meetings, coding, and memo writing	Healthcare providers working with hip fracture patients (*n* = 17)	Dominant themes: a focus on process: information gathering and communication; focus on outcomes: autonomy and care pathways.

Toscan et al., 2013 [54]	Canada	Explore multiple transitions of a single hip fracture patient from multiple perspectives	Interviews, participant observation, analysis of current literature; inductive analysis incorporating data reduction, display, and conclusion drawing	One hip fracture patient (age: 80s) who underwent multiple transitions, her family caregivers, and healthcare providers	Four themes over trajectory related to patients and caregivers not feeling involved in care, confusion over healthcare providers' role, uncertainty, and individualized care hampered by policies.

Unson et al., 2003 [48]	USA	Examine how beliefs about medication and treatments influence selection and adherence	Focus groups; open, selective coding for themes	Racially diverse women ≥60 not on osteoporosis treatment (60–91 YA)	Adherence affected by multiple factors included beliefs about medication safety, costs, treatment goals, belief in physician's competence, and need for treatment.

Wilkins, 2001 [37]	Canada	Understand relations between self-concept and meanings of aging and chronic illness and implications for everyday lives	Interviews; questionnaire; constant comparative method	Women with osteoporosis (54–80 YA; *n* = 28)	Women with confident selves accepted aging and chronic disease; women with contradictory selves denied effects of aging and chronic disease, and women with disparaged selves were resigned to aging and chronic disease.

^a^YA = years of age.
